# Genetic Adaptation of Mesorhizobium Symbionts Associated With Caragana in Northern China Deserts

**DOI:** 10.1002/ece3.73134

**Published:** 2026-02-17

**Authors:** Xiaoxia Yuan, Hua Li, Xiumin Yu, Zhaojun Ji

**Affiliations:** ^1^ College of Life Science and Food Engineering Inner Mongolia Minzu University Tongliao Inner Mongolia P. R. China; ^2^ Key Laboratory of Mongolian Medicine Research and Development Engineering Ministry of Education Tongliao Inner Mongolia P. R. China; ^3^ Inner Mongolia Autonomous Region Engineering Technology Research Center for Prevention and Control of Pathogenic Bacteria in Milk Tongliao Inner Mongolia P. R. China

**Keywords:** *Caragana*, environmental adaptation, membrane transporters, *Mesorhizobium*, nucleotide repair genes, recombination

## Abstract

*Caragana*, a keystone leguminous species dominating arid semi‐fixed deserts in northern China, forms specialized symbiotic nitrogen‐fixing partnerships with *Mesorhizobium*, which are indispensable for sustaining ecosystem function globally. However, the roles of membrane transporters and nucleotide repair genes in conferring survival advantages to desert‐dwelling *Mesorhizobium* across desert environments remained poorly elucidated. Therefore, a total of 68 representative *Mesorhizobium* strains associated with *Caragana*, isolated from five geographically distant areas (A to E) in the desert belt of northern China, were investigated to elucidate the pivotal roles of three membrane transporters (*cysW*, *exoY*, *idhA*) and two nucleotide repair genes (*mutS*, *uvrC*) in microbial adaptation to environmental stress. Phylogenetic analysis results revealed that strains assigned to the same genospecies primarily clustered by genetic lineage rather than geographic origin, with stronger intralineage sequence cohesion observed relative to interregional divergence. Notably, phylogenetic trees of membrane transporter genes, nucleotide repair genes, and core genes showed high topological congruence, underscoring their concerted evolutionary dynamics and shared selective pressures. Furthermore, consistent nucleotide diversity (*π*), low *πN/πS* ratios (<< 1.0) and genetic distance (*Dxy*) across populations indicated that purifying selection predominated in membrane transporters and nucleotide repair genes. Elevated recombination impact (*r/m*) and frequency (*ρ/θ*) revealed that homogenizing gene flow, rather than mutation, was the primary driver of population differentiation enabling rapid adaptation to desert environments.

## Graphical abstract

This study investigates the genetic adaptation of Caragana‐associated Mesorhizobiumin northwest China's semi‐fixed deserts, focusing on membrane transporters and nucleotide repair genes. Despite geographic isolation, populations exhibited striking genetic congruence, driven by homogeneously harsh environments that weakened the distance‐decay relationship. Recombination (rather than mutation) dominated genetic divergence, with the horizontal transfer of adaptive loci (e.g., *cysW*, *uvrC*) enabling rapid trait dissemination, underscoring these genes' roles in desert symbiotic adaptation.

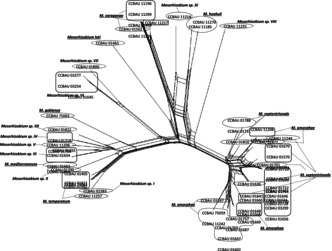

## 1 Introduction

*Caragana* species, pivotal leguminous plants in barren semi‐fixed deserts of northern China, serve dual ecological roles in windbreak/sand fixation and watershed protection. Dominating the arid landscapes of Inner Mongolia, these areas were characterized by strong winds and extreme temperature fluctuations (−30°C to 50°C; Wu et al. 2015). *Caragana*'s remarkable tolerance to the low‐nutrient conditions positioned it as a keystone species for desert ecosystem restoration (He et al. 2024; Li et al. 2014). This keystone role is owing to its symbiotic association with rhizosphere microbes, which enhance soil fertility and nutrient bioavailability, thereby bolstering *Caragana*'s growth and survival while underpinning broader ecological recovery in degraded desert ecosystems (Liu et al. 2022). Among these microbes, *Mesorhizobium* stood out as the primary symbiotic microsymbiont, forming mutualistic relationships with *Caragana* to secure sufficient nitrogen nutrition (Ji et al. 2015). Notably, *Mesorhizobium* exhibited superior alkaline tolerance compared to other rhizobia, a trait attributed to its capacity to secrete organic acids, enabling colonization and N_2_ fixation in the alkaline soils typical of *Caragana* habitats (Chen et al. 2010). This adaptation was pivotal, enabling *Mesorhizobium* to thrive in *Caragana*'s root zone and establish a robust symbiosis that enhanced plant resilience in harsh environments.

The *Caragana‐Mesorhizobium* symbiosis balanced mutual benefits (*Caragana* gains nitrogen, *Mesorhizobium* gains carbon) with energetic costs to the plant. Nevertheless, the fitness gains for both organisms justified this mutualism as the evolutionarily stable strategy (Carlson and Frederickson 2023; Mathesius 2022). Various *Mesorhizobium* species, including *M. amorphae* , *M. septentrionale* , *M. caraganae* , *M. gobiense* , *M. temperatum* , *M. mediterraneum* , *M. shangrilense* , *M. tianshanense* , *M. huakuii* , and *M. metallidurans* , have been documented to nodulate *Caragana* in extreme arid/semi‐arid deserts with fluctuating temperatures (Liu et al. 2022; Yan et al. 2017).

The ecological persistence and environmental adaptation of *Mesorhizobium* symbionts associated with *Caragana* in northern China deserts depend on pivotal genetic systems. Specifically, membrane transporter genes such as *cysW* (encoding a cysteine ABC transporter subunit, critical for sulfur assimilation and cysteine uptake to support redox balance; Chakraborty et al. 2021; Li et al. 2025), *exoY* (involved in exopolysaccharide biosynthesis, facilitating biofilm formation and adhesion to root surfaces; Jiang et al. 2001; Sakthivel et al. 2025), and *idhA* (encoding isocitrate dehydrogenase, a key enzyme in the tricarboxylic acid cycle that sustains energy production under nutrient limitation; Yang et al. 2022) were essential for adapting to nutrient‐poor and alkaline soils. Concurrently, nucleotide repair genes like *mutS* (mediating mismatch repair to correct replication errors and maintain genomic integrity; Margara et al. 2016; Martín‐Blecua et al. 2025) and *uvrC* (participating in nucleotide excision repair to counteract DNA damage from UV radiation or reactive oxygen species; Najjari et al. 2023; Santoyo et al. 2005) ensured genetic stability in harsh environments. Together, these genes underpinned the adaptive plasticity of *Mesorhizobium*, enabling it to thrive in extreme desert conditions and sustain its symbiotic relationship with *Caragana*. Therefore, adaptive strategies in *Caragana*‐nodulating *Mesorhizobium* would be investigated in this study, with a focus on genetic diversity, recombination, and gene flow mediated by membrane transporter genes (*cysW*, *exoY*, *idhA*) and nucleotide repair genes (*mutS*, *uvrC*) for clarifying the adaptation of symbiotic rhizobia to extreme environments.

## 2 Materials and Methods

### 2.1 Rhizobial Strains and Gene Sequencing

A total of 708 mesorhizobial strains isolated from the roots of *Caragana* distributed across five desert areas (Areas A–E, three sampling sites in Areas A, C, D; four in Area B; and six in Area E) along the semi‐fixed desert belt in northern China (data not shown). And 68 representatives were selected based on the phylogenetic tree of the *recA* gene for analysis (Table S1). Nine type strains including *Mesorhizobium septentrionale* SDW 014^T^, *M. gobiense* CCBAU 83330^T^, *M. amorphae* ACCC 19665^T^, *M. mediterraneum* USDA 3392^T^, *M. tianshanense* CCBAU 3306^T^, *M. metallidurans* LMG 24485^T^, *M. temperatum* SDW018^T^ and *M. huakuii* CCBAU 2609^T^ and *M. caraganae* CCBAU 11299^T^ were employed as references to establish the phylogenetic relationship of these *Caragana*‐nodulating mesorhizobia. All strains were cultured in YMA medium at 28°C and preserved in 30% glycerol at −80°C following established protocols.

### 2.2 DNA Extraction and Gene Sequencing

The total template DNA of these *Caragana* mesorhizobia was extracted using the method as described previously (Terefework et al. 2001). The biological function and PCR amplification protocols were shown in Table S2 for nine genes, including three membrane transporter genes *cysW* (Sirko et al. 1995), *exoY* (Reed et al. 1991), *idhA* (Jiang et al. 2001), and two nucleotide repair genes *mutS* (Martínez‐Salazar et al. 2009), *uvrC* (Cubo et al. 1992), two core genes *recA*, *rpoB*, and two nodulation genes *nodC*, *nodD*. The primers were designed by using Premier 5.0 software based on conserved regions of *cysW*, *exoY*, *idhA*, *mutS* and *uvrC*, identified from whole‐genome sequences of the reference *Mesorhizobium* strains (CCBAU01583, CCBAU01570, CCBAU01502, CCBAU01399), as detailed in Table S2. And the PCR products were purified and commercially sequenced by ABI 3730xl sequenator in Beijing, China. Then, the sequences were manually checked using Chromas Pro (Ver. 2.1.3) and edited manually using DNAMAN (Ver. 9.0). Total 340 nucleotide sequences of 5 genes *cysW*, *exoY*, *idhA*, *mutS* and *uvrC* were newly obtained in this study which were deposited in the GenBank database under accession numbers MK252304 through MK252643 (Table S4).

### 2.3 Phylogenetic Analyses

These nucleotide sequences were aligned and the redundant bases removed using the ClustalW program. MEGA11.0 program was used for constructing the Neighbor‐Joining (NJ) phylogenetic trees for membrane transporters, nucleotide repair, core, and nodulation genes with the Kimura‐2‐parameter model (1000 bootstrap replicates) (Tamura et al. 2011), respectively. Then, reticulate evolutionary events, including potential hybridization and recombination, were inferred by quantifying phylogenetic network complexity using SPLITSTREE 4.0 software with the Neighbor‐Net algorithm (1000 bootstrap replicates; Huson and Bryant 2006).

### 2.4 Nucleotide Polymorphism and Population Genetics Analyses

Nucleotide polymorphisms including the haplotype diversity (*Hd*), number of haplotypes (*h*), nucleotide diversity (*π*), the *π*_*N*_/*π*_*S*_ ratios (where *πS* denotes synonymous substitutions per synonymous site and *πN* denotes non‐synonymous substitutions per non‐synonymous site) (Nei and Gojobori 1986), and two additional metrics: *Dxy* (average nucleotide divergence) and *Nm* (gene flow estimated as migrants) were estimated using DnaSP v5 software (Librado and Rozas 2009). STRUCTURE software was employed to analyze admixture levels in *Mesorhizobium* genospecies under the LOCPRIOR model (100,000 burn‐in and 1,000,000 iterations), with *K* (number of ancestral subpopulations) estimated separately for membrane transporters and nucleotide repair genes (Hubisz et al. 2009; Jakobsson and Rosenberg 2007).

### 2.5 Recombination Estimation

The minimal recombination events (*Rm*) within the populations were estimated using the DnaSP v5 (Hudson and Kaplan 1985). Two recombination rates—*r/m* (the relative impact of recombination compared with that of point mutation in lineage genetic diversification) and *ρ/θ* (the relative frequency of recombination occurrence compared with that of point mutation in lineage history), were calculated. The effect of recombination on evolutionary history was deduced using CLONALFRAME with proper parameters (100,000 burn‐in iterations plus 1,000,000 sampling iterations per run) for three independent runs (Didelot and Falush 2007).

## 3 Results

### 3.1 Phylogenies and Nucleotide Polymorphisms of *Caragana* Mesorhizobia

**FIGURE 1:**
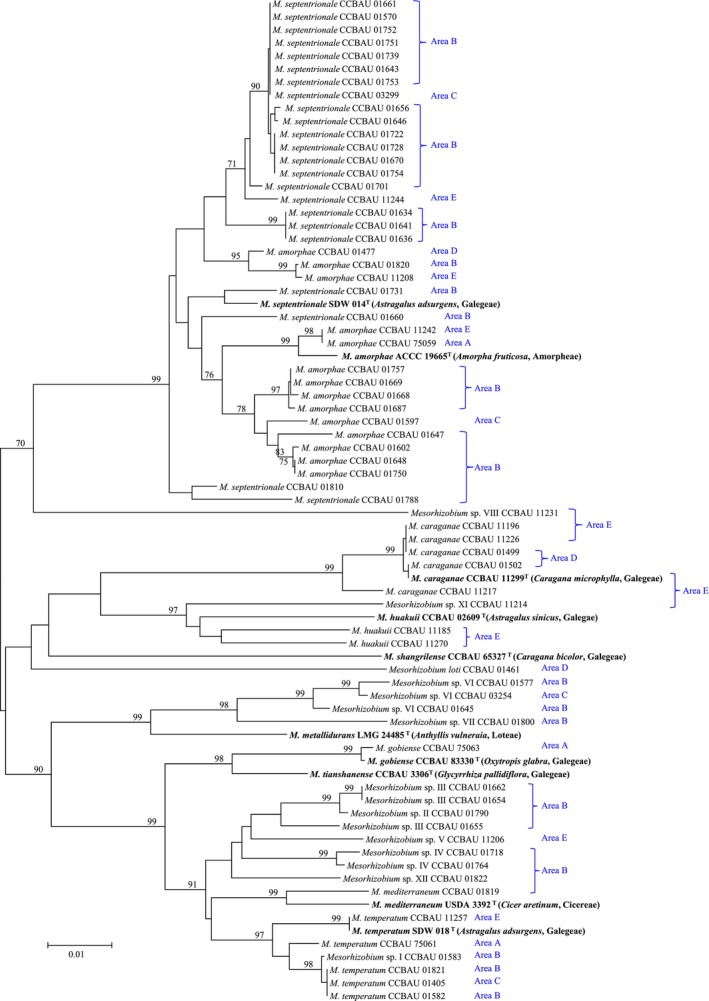
Neighbor‐Joining (NJ) tree constructed based upon the concatenated sequences of membrane transporters. Three membrane transporter genes (*cysW*, *exoY*, *idhA*) were used. Bootstrap values greater than 70% are indicated at the branch points. The type strains (Bold Fonts) are shown in parentheses after the strain numbers. The scale bar represents 1% nucleotide substitutions.

**FIGURE 2:**
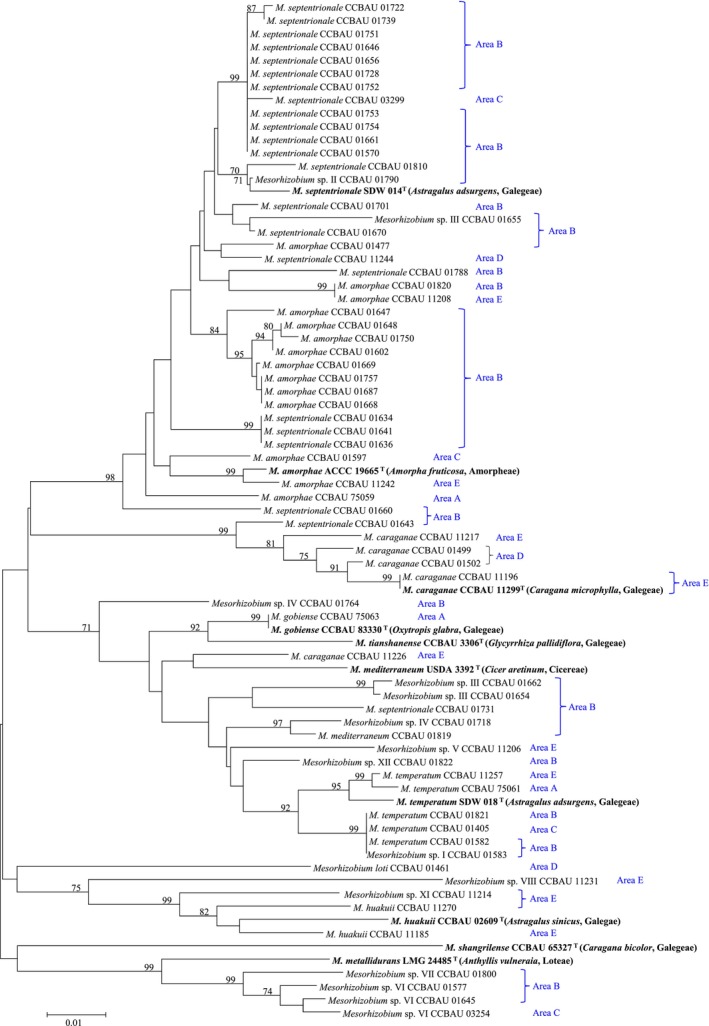
Neighbor‐Joining (NJ) tree constructed based upon the concatenated sequences of nucleotide repair genes. Two nucleotide repair genes (*mutS*, *uvrC*) were used. Bootstrap values greater than 70% are indicated at the branch points. The type strains (Bold Fonts) are shown in parentheses after the strain numbers. The scale bar represents 1% nucleotide substitutions.

These representative *Caragana*‐associated mesorhizobia, isolated from different areas (A–E), were distributed across different branches, without geographical isolation across five distinct areas of the semi‐fixed desert belt in northern China. The dominant mesorhizobial communities of areas (A–E) comprised genospecies *M. amorphae* and *M. septentrionale* , which also exhibited a closely genetic relationship on the phylogenetic trees of membrane transporters (Figure 1), nucleotide repair genes (Figure 2), and core genes (Figure S1). Notably, the genospecies *M. amorphae* encompassed representative strains of *Mesorhizobium* sampled from all five desert areas (A–E) with an extensive genetic admixture and cross‐regional dispersal. This adaptation was primarily mediated by the convergent evolution of key genes, including membrane transporters (*cysW*, *exoY*, *idhA*) and nucleotide repair genes (*mutS*, *uvrC*), which overcame geographic barriers. In addition, the topological structures of the phylogenetic trees were similar to each other based on the concatenated sequences of membrane transporters, nucleotide repair genes, and core genes, although some variations were observed in specific cases (e.g., CCBAU 01790, CCBAU 01655, CCBAU 11226, and CCBAU 01731). However, the phylogenetic tree based on nodulation genes (*nodC*, *nodD*) exhibited a markedly different topological structure (Figure S2), although these mesorhizobial strains cross‐dispersed across five areas branched into multiple clades.

Nucleotide polymorphisms for each gene were presented in Table 3. No significant differences were detected in haplotype number (*h*) and haplotype diversity (*Hd*) values across the tested genes. Notably, the average nucleotide diversity (*π*) value of nodulation genes (0.03501) was markedly lower than that of membrane transporters (0.06797), nucleotide repair genes (0.07449), and core genes (0.05498), highlighting the particularity of symbiotic adaptation. It is noted that the *πS* values (synonymous substitutions per site) were significantly higher than *πN* values (non‐synonymous substitutions per site) for all tested genes. The *πN/πS* values were significantly higher for *nodC* and *nodD* than for other genes. In addition, *π* and *πN/πS* values of these strains from all five areas (A–E) were consistent for core, membrane transporter, and nucleotide repair genes, respectively (Table 4).

### 3.2 Gene Flow and Genetic Divergence of *Caragana*‐Associated Mesorhizobia

**FIGURE 3:**
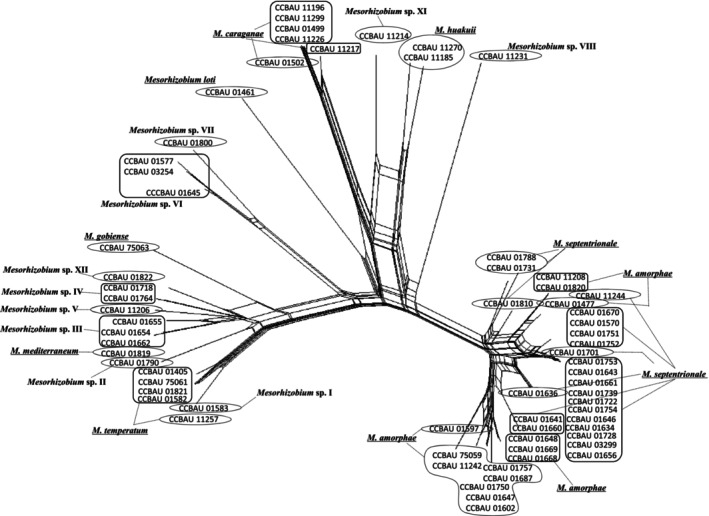
The Neighbor‐Nets generated using SplitsTree4 with the Hamming distance option. The trees are constructed based upon the concatenated membrane transporter genes (*cysW*, *exoY*, *idhA*). The scale bar represents 1% nucleotide substitutions.

**FIGURE 4:**
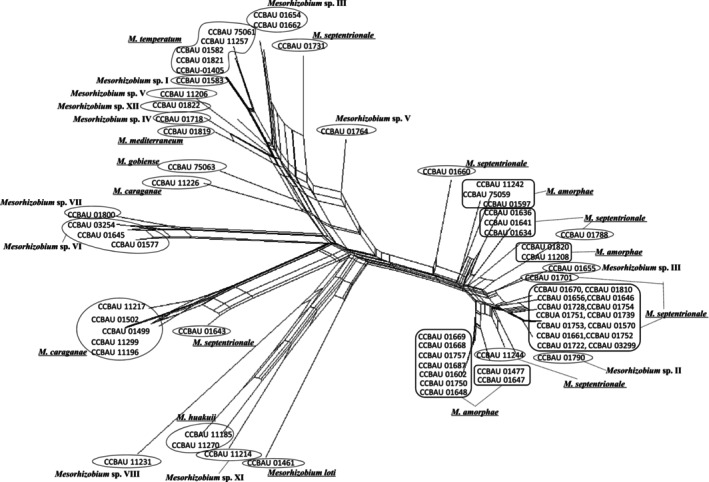
The Neighbor‐Nets generated using SplitsTree4 with the Hamming distance option. The trees are constructed based upon the concatenated nucleotide repair genes (*mutS*, *uvrC*). The scale bar represents 1% nucleotide substitutions.

**FIGURE 5:**
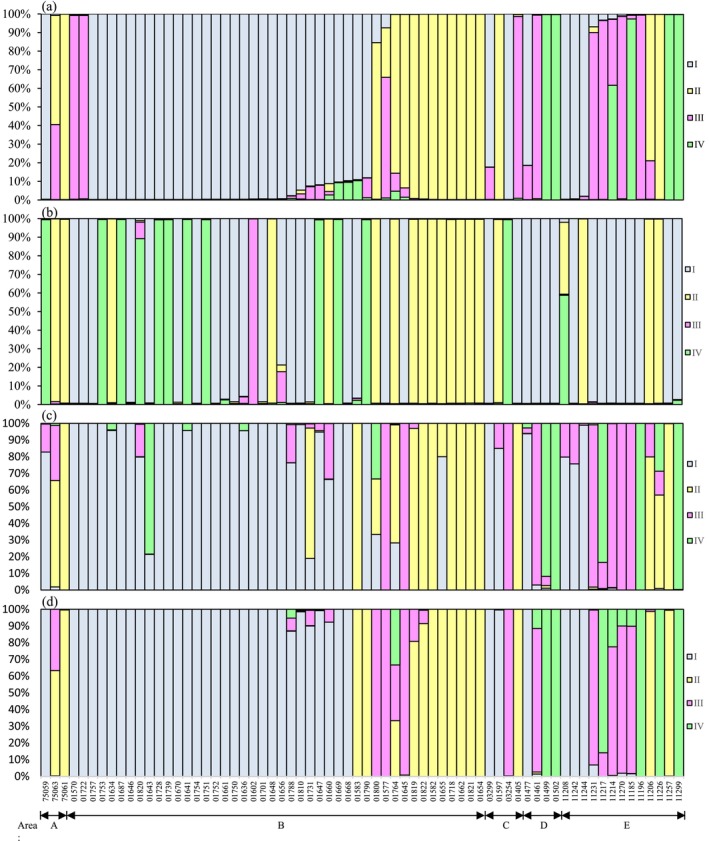
STRUCTURE analyses of *Caragana*‐associated *Mesorhizobium* populations from different areas. Concatenated core (a), nodulation (b), membrane transporter (c) and nucleotide repair (d) genes were analyzed. The inferred ancestries are designated sequentially as I, II, III, and IV, shown in bars and filled with different grayness or dots. The horizontal axis represents current *Mesorhizobium* individuals (in the same order in all panels), and the bar for each individual is filled according to the inferred proportions of single‐nucleotide alleles that were derived from each of the ancestries. The vertical axis represents the percentage of the strains.

NeighborNet phylogenetic network analysis revealed a complex, reticulate recombination network architecture in both membrane transporter and nucleotide repair gene datasets (Figures 3 and 4), indicative of pervasive recombination‐driven gene flow and genetic exchange among *Caragana*‐associated mesorhizobia across the five desert areas (A–E) in northern China. In addition, four distinct genetic lineages (I, II, III, and IV) were identified by STRUCTURE analysis (Figure 5). Notably, lineage I was ubiquitously distributed across all five areas and served as the primary ancestral source in Area B. Lineages II–IV, by contrast, displayed irregular, patchy distributions, likely reflecting localized adaptation or stochastic recombination events. Critically, most strains (e.g., CCBAU 75063 and CCBAU 01764) showed evidence of mixed ancestry, inheriting genetic material from two or more lineages (Figure 5).

Genetic differentiation (quantified as pairwise *Dxy*) among rhizobial populations isolated from the five desert areas (A–E) exhibited no statistically significant variation (Table 1). Notably, both the mean genetic distance (*Dxy*) and nucleotide diversity (*π*) values remained comparable across all areas (Tables 1 and 4), reflecting consistent levels of intra‐population genetic variation despite geographic dispersion. Dxy values, calculated from the concatenated membrane transporter genes, ranged from a minimum of 0.06215 (between areas B and C) to a maximum of 0.09687 (between areas A and D).

**TABLE 1:** Genetic divergence (presented as *Dxy*) and gene flow (presented as *Nm*) of *Caragana*‐associated *Mesorhizobium* isolated from five areas (A–E).

*Nm* *Dxy*	A	B	C	D	E
Concatenated membrane transporters					
A		22.07	4.02	2.72	7.35
B	0.07078^e^		5.10	1.89	2.81
C	0.07228^e^	0.06215^e^		0.82	11.21
D	0.09687^e^	0.08627^e^	0.09222^e^		9.92
E	0.09052^e^	0.08380^e^	0.08823^e^	0.07954^e^	
Concatenated nucleotide repair genes					
A		18.17	4.10	8.55	18.84
B	0.07013^e^		6.76	4.30	3.82
C	0.07317^e^	0.06594^e^		0.02	36.76
D	0.08771^e^	0.07896^e^	0.08582^e^		16.93
E	0.08238^e^	0.08199^e^	0.08639^e^	0.08453^e^	
Concatenated core genes					
A		96.64	−5.53	3.47	27.05
B	0.05465^e^		−7.09	2.87	6.68
C	0.05631^e^	0.05097^e^		9.63	−22.15
D	0.06627^e^	0.05970^e^	0.06143^e^		−32.35
E	0.06269^e^	0.05856^e^	0.06062^e^	0.05715^e^	
Concatenated nodulation genes					
A		94.64	−5.53	3.47	27.05
B	0.00029^e^		−7.09	2.87	6.68
C	0.05631^e^	0.05097^e^		9.63	−22.15
D	0.06627^e^	0.05970e	0.06143^e^		−32.35
E	0.06269^e^	0.05970^e^	0.06062^e^	0.05715^e^	

*Note:* Number of gene flow (*Nm*) and average nucleotide divergence between groups (*Dxy*) are shown in the upper and lower triangular of the table. Statistical difference letter is marked as superscript after each number in the lower triangular of the table: e, non‐significant.

### 3.3 Recombination Events Among the *Caragana* Mesorhizobial Lineages

A total of 61 and 57 minimal recombination events (*Rm*) were inferred using DnaSP v5 software for the concatenated membrane transporter and nucleotide repair genes, respectively (Table 2). Subsequent analysis with CLONALFRAME revealed contrasting evolutionary dynamics: membrane transporter genes exhibited a higher recombination impact (*r/m* = 2.48) and a higher frequency (*ρ/θ* = 0.72) compared to nucleotide repair genes (*r/m* = 1.15; *ρ/θ* = 0.33). Notably, *r/m* values were > 1.0 for both gene categories, showing that recombination, not mutation, was the dominant driver of genetic diversification. This evolutionary process was also corroborated by SH tests, which showed significant topological incongruence between gene trees of membrane transporter/nucleotide repair loci and the core gene‐derived species tree (*p* < 0.05; Table S3).

**TABLE 2:** Recombination analysis of *Caragana*‐associated *Mesorhizobium*.

Genes	Length (bp)	*Rm*	*r/m*	*ρ/θ*
Concatenated membrane transport genes	837	61	2.48	0.72
Concatenated nucleotide repair genes	705	57	1.15	0.33
Concatenated core genes	879	57	21.46	6.78
Concatenated nodulation genes	999	62	171.39	63.22

*Note:* *Rm*, observed minimum number of recombination events; *r/m*, the relative impact of recombination compared with that of point mutation in the genetic diversification of the lineage; *ρ/θ*, the relative frequency of the occurrence of recombination compared with that of point mutation in the history of the lineage.

**TABLE 3:** Molecular diversity for these genes of *Caragana*‐associated *Mesorhizobium*.

Genes	Length (bp)	*S*	*Eta*	*h/Hd*	*π*	*π*_*S*_	*π*_*N*_	*π*_*N*_ */π*_*S*_
Membrane transporters								
*cysW*	327	100	129	35/0.944	0.06547	0.23925	0.01569	0.06558
*exoY*	300	99	141	40/0.970	0.09122	0.47662	0.01149	0.02411
*idhA*	210	52	69	35/0.942	0.04722	0.20279	0.00921	0.04542
Avg.	279	83.67	113.0	36.7/0.952	0.06797	0.30622	0.01213	0.04503
Nucleotide repair genes								
*mutS*	144	54	68	36/0.935	0.08291	0.35343	0.01814	0.05133
*uvrC*	561	173	227	45/0.974	0.06607	0.25698	0.01782	0.06934
Avg.	352.5	113.5	147.5	40.5/0.955	0.07449	0.30521	0.01798	0.06033
Core genes								
*recA*	288	75	98	49/0.983	0.05502	0.23468	0.00154	0.00656
*rpoB*	591	179	219	46/0.958	0.05494	0.19047	0.02090	0.10973
Avg.	439.5	127	158.5	47.5/0.971	0.05498	0.21258	0.01122	0.05815
Nodulation genes								
*nodC*	507	131	149	30/0.912	0.02718	0.07595	0.01185	0.15602
*nodD*	492	119	132	71/1.000	0.04283	0.10580	0.02666	0.25198
Avg.	499.5	125	140.5	50.5/0.956	0.03501	0.09088	0.01926	0.20400

*Note:* *S*, segregating sites or number of polymorphic (segregating) sites; *Eta*, total number of mutations. *h*, haplotype number; *Hd*, haplotype diversity; *π*, average number of nucleotide differences per site between two sequences; *π*_*S*_, nucleotide diversity for synonymous substitutions; *π*_*N*_, nucleotide diversity for nonsynonymous substitutions.

**TABLE 4:** Nucleotide polymorphism of five areas (A–E) for these genes of *Caragana*‐associated *Mesorhizobium*.

Area (No. of strains)	Length (bp)	*S*	*Eta*	*h*/*Hd*	*π*	*π*_*S*_	*π*_*N*_	*π*_*N*_/*π*_*S*_
Concatenated membrane transporters								
A (3)	837	102	106	3/1.000	0.08284	0.37023	0.01139	0.03076
B (44)	837	184	224	29/0.964	0.05559	0.23325	0.00926	0.03970
C (4)	837	119	131	4/1.000	0.08224	0.34824	0.01523	0.04373
D (4)	837	116	127	4/1.000	0.08084	0.35292	0.01457	0.04128
E (13)	837	196	243	12/0.987	0.08668	0.38097	0.01543	0.04050
Average	—	143.4	166.2	10.4/0.990	0.07764	0.33712	0.01318	0.03920
Concatenated nucleotide repair genes								
A (3)	705	83	87	3/1.000	0.08038	0.30316	0.02337	0.07709
B (44)	705	171	206	30/0.952	0.05612	0.21654	0.01412	0.06521
C (4)	705	107	114	4/1.000	0.08629	0.34927	0.02238	0.06408
D (4)	705	102	114	4/1.000	0.08534	0.35791	0.02154	0.06018
E (13)	705	168	204	12/0.987	0.08887	0.35717	0.02370	0.06635
Average	—	126.2	145	10.6/0.988	0.07940	0.31681	0.02102	0.06658
Concatenated core genes								
A (3)	879	79	83	3/1.000	0.06143	0.18865	0.01897	0.10055
B (44)	879	174	199	34/0.978	0.04729	0.17233	0.01297	0.07526
C (4)	879	96	101	4/1.000	0.06238	0.19755	0.01709	0.08651
D (4)	879	75	98	49/0.983	0.05502	0.17007	0.01436	0.08443
E (13)	879	174	193	13/1.000	0.06167	0.19369	0.01737	0.08967
Average	—	119.6	134.8	20.6/0.992	0.05756	0.18446	0.01615	0.08728
Concatenated nodulation genes								
A (3)	999	74	75	3/1.000	0.03313	0.08849	0.03634	0.41066
B (44)	999	182	196	44/1.000	0.02718	0.07962	0.01712	0.21502
C (4)	999	46	46	4/1.000	0.02536	0.05962	0.01348	0.22609
D (4)	999	20	20	4/1.000	0.01118	0.02938	0.00493	0.16780
E (13)	999	124	130	13.1/1.000	0.03722	0.08425	0.02106	0.24997
Average	—	89.2	93.4	13.62/1.000	0.02681	0.06827	0.01859	0.25391

*Note:* *S*, segregating sites or number of polymorphic (segregating) sites; *Eta*, total number of mutations. *h*, haplotype number; *Hd*, haplotype diversity; *π*, average number of nucleotide differences per site between two sequences; *π*_*S*_, nucleotide diversity for synonymous substitutions; *π*_*N*_, nucleotide diversity for nonsynonymous substitutions. —, null.

## 4 Discussion

### 4.1 Phylogenies and Genetic Divergence of *Caragana* Mesorhizobia Based on Membrane Transporters and Nucleotide Repair Genes

The phylogenetic congruence observed across membrane transporters and nucleotide repair genes (Figures 1 and 2) revealed an evolutionary conservatism pattern of *Caragana*‐symbiotic *Mesorhizobium*, with parallel adaptive trajectories in response to shared environmental pressures (Ament‐Velásquez et al. 2022; Yamamoto et al. 2021), despite geographic isolation across northern China's semi‐fixed deserts (spanning > 1000 km). The intertwined phylogenetic patterns indicated that environmental selection pressures likely dominated the genetic adaptation of these strains (Ji et al. 2020). The contrasting topologies observed in nodulation gene trees highlighted the divergent evolutionary trajectories of symbiosis‐related traits, which are likely shaped by host‐specific selection pressures (Provorov et al. 2022). In contrast, the conserved topologies of membrane‐transporter and nucleotide repair gene trees (Figures 1 and 2) suggested that these loci were under strong purifying selection to maintain basal physiological functions critical for desert survival (Karasev et al. 2023). This phylogenetic cohesion contrasted sharply with the divergence observed in nodulation genes (*nodC*, *nodD*), suggesting that membrane transporters and DNA repair enzymes operated under distinct selective regimes (Karasev et al. 2023; Liu et al. 2023). Therefore, the strong selection pressure (pH > 8.0, K^+^ content shown in Table S1) in the semi‐fixed desert belt enhanced the compositional similarity index of these *Mesorhizobium* strains and greatly weakened their distance‐decay relationship, one of the most well‐documented universal biogeographic patterns in natural ecosystems (Chen et al. 2016; Ji et al. 2015; Zhang et al. 2020). Therefore, in the dynamic environmental conditions of northern China's semi‐fixed deserts, *Mesorhizobium* strains exhibit a striking evolutionary signature characterized by strong purifying selection alongside rapid adaptive evolution, reflecting their dual response to abiotic stress homogenization and niche‐specific innovation (Teng et al. 2023).

The uniform nucleotide diversity (*π*) and low *πN*/*πS* ratios (< 1.0) across all populations (Table 4) indicated that pervasive purifying selection might act on membrane transporters and nucleotide repair genes. These genes encode critical functions for desert survival: *cysW* mediates sulfate uptake for osmotic regulation (Srivastava et al. 2025), *uvrC* repairs UV‐induced DNA lesions (Selby 2017), while *idhA* modulates carbon flux under nutrient limitation (Lin et al. 2022). No significant difference in genetic divergence (*Dxy*) values among *Caragana*‐symbiotic *Mesorhizobium* from geographically distinct areas (Table 1) was observed, inferring that these strains might be canalized by abiotic stressors, such as extreme temperatures (−30°C to 50°C) (Peters et al. 2026; Rychel et al. 2025), alkaline soils (pH 8.5–10.2) (Shah et al. 2022), and saline‐alkali gradients (Fall et al. 2019), to maintain physiological homeostasis, and environmental selection might be the dominant driver.

In contrast to the divergence observed in nodulation genes (Wendlandt et al. 2021), the high gene flow and recombination rates among membrane transporter genes (Table 2) indicated that these genes were subject to host selection for desert‐adapted *Mesorhizobium*. This was exemplified by lineage I, which dominated in all five areas (Figure 5) and carried a suite of broadly adaptive genes. Such genomic fluidity, driven by recombination, allowed rapid dissemination of beneficial mutations, and ensured metabolic robustness across fragmented desert habitats, confirming that recombination was an essential agent of adaptive homogenization for *Caragana*‐symbiotic *Mesorhizobium* (Good et al. 2025; Mazzamurro et al. 2025).

While symbiosis‐related genes (*nodC*, *nodD*) exhibited elevated *πN/πS* ratios, reflecting host‐driven diversification (Singh and Valdés‐López 2023), the stoichiometric stability of membrane transporters and DNA repair enzymes underscored their role as evolutionary “buffer zones”(Moger‐Reischer et al. 2023). By maintaining core physiological functions, these genes relieved selective constraints on symbiosis loci, allowing niche‐specific innovations to evolve independently (Forni et al. 2025). This modular evolutionary architecture, where housekeeping (core) genes prioritized environmental adaptation and symbiosis genes prioritized host specialization, might underpin the resilience of desert rhizobia‐legume mutualisms, reflecting the implications for symbiosis‐environment coevolution.

### 4.2 Gene Flow, Recombination, and Environmental Adaptation of *Caragana* Mesorhizobia

The gene flow among all mesorhizobia, especially between the dominant genospecies *M. septentrionale* and *M. amorphae* associated with *Caragana*, was frequent for all tested genes (Figures S1 and S2), indicating that genetic diversity between coexisting strains of the same genospecies or closely related genospecies is an important ecological mechanism explaining the diversity of *Caragana* mesorhizobia (Fields et al. 2022). Extensive gene flow, characterized by elevated *Nm* values (Table 1), was observed across *Caragana*‐associated *Mesorhizobium* populations. Notably, membrane transporters exhibited particularly high gene flow between areas A and B (*Nm* = 22.07), whereas nucleotide repair genes showed pronounced gene flow between areas C and E (*Nm* = 36.76). These patterns underscored the dominant role of gene recombination in shaping the evolutionary trajectories of these symbionts. Complex gene interweaving, clearly found among many strains isolated from areas A to E (Figure 5), also indicated that *Caragana*‐associated *Mesorhizobium* populations experienced similar selection pressures, although they alternated between semi‐fixed desert soil and host environments (Burghardt et al. 2019). The high relative impact (*r/m*) and frequency (*ρ/θ*) of recombination for concatenated membrane transporter genes (Table 2) also indicated that gene recombination had played an important role in the evolutionary history. The strains tended to acquire special transporters in membranes for nutrient acquisition via recombination in various hostile environments, enhancing the evolutionary stability of nodulation on their hosts (de Faria et al. 2022). This pattern was particularly evident in membrane transporters (*cysW*, *exoY*, *idhA*), which exhibited the highest recombination frequencies (*r/m* = 2.48), underscoring their adaptive function across desert landscapes. In contrast, nucleotide repair genes (*mutS*, *uvrC*) maintained stricter purifying selection (*πN/πS* < 1), ensuring genome integrity under extreme abiotic stress (Lin et al. 2007). A selection‐driven recombination paradigm revealed that membrane transporters prioritized functional stability over sequence divergence. For example, recombination hotspots in the *cysW* gene spanned substrate‐binding domains, optimizing sulfate uptake efficiency under fluctuating salinity (Xamxidin et al. 2025), and nucleotide repair genes preserved symbiotic fidelity by suppressing non‐synonymous substitutions, enabling host‐specific nodulation traits to evolve independently, such as *uvrC* assisting in the adaptive piecing‐together of UV repair modules (Springall et al. 2018). This dual strategy, functional optimization via recombination for membrane genes and functional optimization via purifying selection for repair genes, enabled simultaneous adaptation to abiotic stress and maintenance of symbiotic precision. The congruent phylogenetic patterns of membrane transporters and nucleotide repair genes (Figures 1 and 2) further contrasted with the divergence of symbiosis‐related loci (*nodC*, *nodD*), revealing a modular evolutionary architecture where core genes buffer physiological stress.

Collectively, these findings established that recombination, rather than geographic isolation, represented the predominant evolutionary force shaping the adaptive trajectories of membrane transporters and nucleotide repair genes, thereby conferring ecological resilience to *Caragana*‐associated *Mesorhizobium* across heterogeneous desert landscapes. However, quantitative integration of environmental variables (e.g., soil pH, salinity) with genetic data lacked here. Future studies should employ systematic sampling and eco‐genomic approaches to disentangle abiotic drivers of adaptation.

## 5 Conclusion

The evolutionary trajectories of *Caragana*‐associated *Mesorhizobium* populations exhibited striking congruence in membrane transporters and nucleotide repair genes, reflecting adaptive convergence shaped by the harsh desert environment. Despite originating from geographically distinct areas (A–E) across the semi‐fixed desert belt in northern China, these rhizobia demonstrated synchronized evolutionary patterns, likely driven by homogenizing selective pressures, highlighting the dominance of environmental homogeneity over geographic isolation in structuring microbial communities. Genetic divergence among populations arose primarily via recombination, rather than mutation, with extensive recombination disseminating adaptive loci. Our findings illuminated the concerted evolutionary dynamics and shared selective pressures of microbial resilience in extreme ecosystems. Future studies should integrate functional genomics to unravel the adaptive roles of these genes and explore synthetic biology approaches to engineer stress‐resilient microbial populations.

## Author Contributions

**Xiaoxia Yuan:** investigation (lead), writing – original draft (lead). **Hua Li:** funding acquisition (supporting), project administration (supporting). **Xiumin Yu:** data curation (equal). **Zhaojun Ji:** funding acquisition (supporting), project administration (equal), writing – review and editing (lead).

## Funding

This study was financially supported by Natural Science Foundation of Inner Mongolia Autonomous Region of China (2024JQ12), National Natural Science Foundation of China (32170020), and Key Research and Development Program of Inner Mongolia Autonomous Region of China (2025YFDZ0116).

## Conflicts of Interest

The authors declare no conflicts of interest.

## Supporting information


**FIGURE S1:** Neighbor‐Joining (NJ) tree constructed based upon the concatenated sequences of core.
**FIGURE S2:** Neighbor‐Joining (NJ) tree constructed based upon the concatenated sequences of nodulation genes.
**TABLE S1:** A total of 68 representative strains isolated from *Caragana*‐associated *Mesorhizobium* used in this study.
**TABLE S2:** Primers designed for the genes and the annealing temperature of them (Tm, °C) in PCR amplification.
**TABLE S3:** Shimodaira‐Hasegaewa (SH) test of each test gene locus in comparison with the concatenated core genes.
**TABLE S4:** Accession numbers of the genes obtained in this study and deposited in GenBank.

## Data Availability

The DNA sequences generated in this study have been deposited in the National Center for Biotechnology Information (NCBI) database. The accession numbers are available in the Table S4. All required data have been uploaded as Supporting Information S1.
