# Supplementary material for: Genetic Adaptation of Mesorhizobium Symbionts Associated With Caragana in Northern China Deserts
Source: Ecol Evol. 2026 Feb 17;16(2):e73134. doi: 10.1002/ece3.73134 (PMC12912947; doi:10.1002/ece3.73134)
Supplement: Supplementary file 1 — FIGURE S1: Neighbor‐Joining (NJ) tree constructed based upon the concatenated sequences of core. FIGURE S2: Neighbor‐Joining (NJ) tree constructed based upon the concatenated sequences of nodulation genes. TABLE S1: A total of 68 representative strains isolated from Caragana‐associated Mesorhizobium used in this study. TABLE S2: Primers designed for the genes and the annealing temperature of them (Tm, °C) in PCR amplification. TABLE S3: Shimodaira‐Hasegaewa (SH) test of each test gene locus in comparison with the concatenated core genes. TABLE S4: Accession numbers of the genes obtained in this study and deposited in GenBank. [file ECE3-16-e73134-s001.pdf]

Supplementary materials

Yuan X.X., Li H., Yu XM., Ji Z.J.*. Genetic Adaptation of *Mesorhizobium* Symbionts associated with *Caragana* in Northern China Deserts.

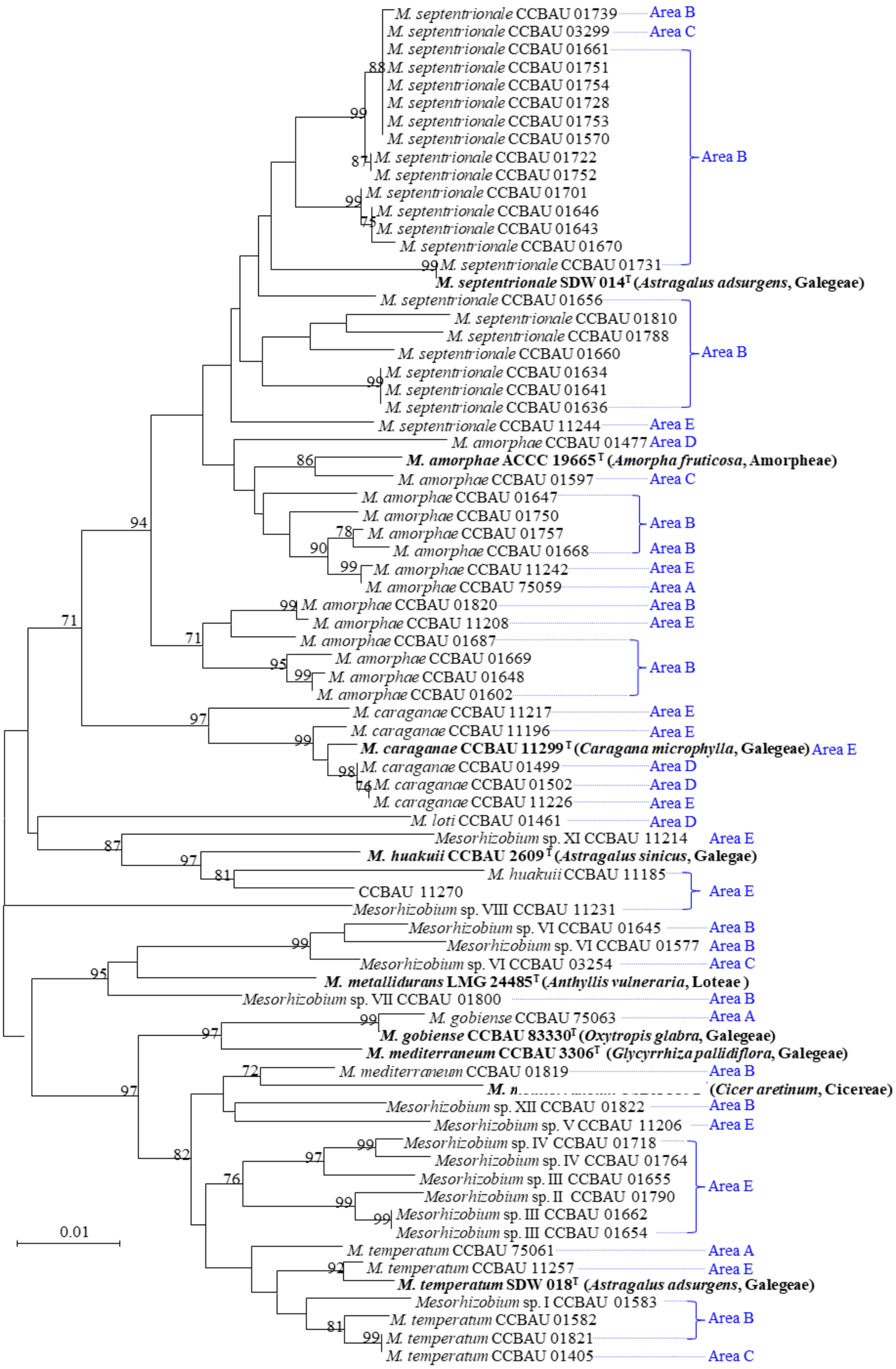

Supplementary Fig. S1. Neighbor-Joining (NJ) tree constructed based upon the concatenated sequences of core genes. Two core genes (*recA*, *rpoB*) were used. Bootstrap values greater than 70% are indicated at the branch points. The host plants of the type strains (Bold Fonts) are shown in parentheses after the strain numbers. The scale bar represents 1% nucleotide substitutions.

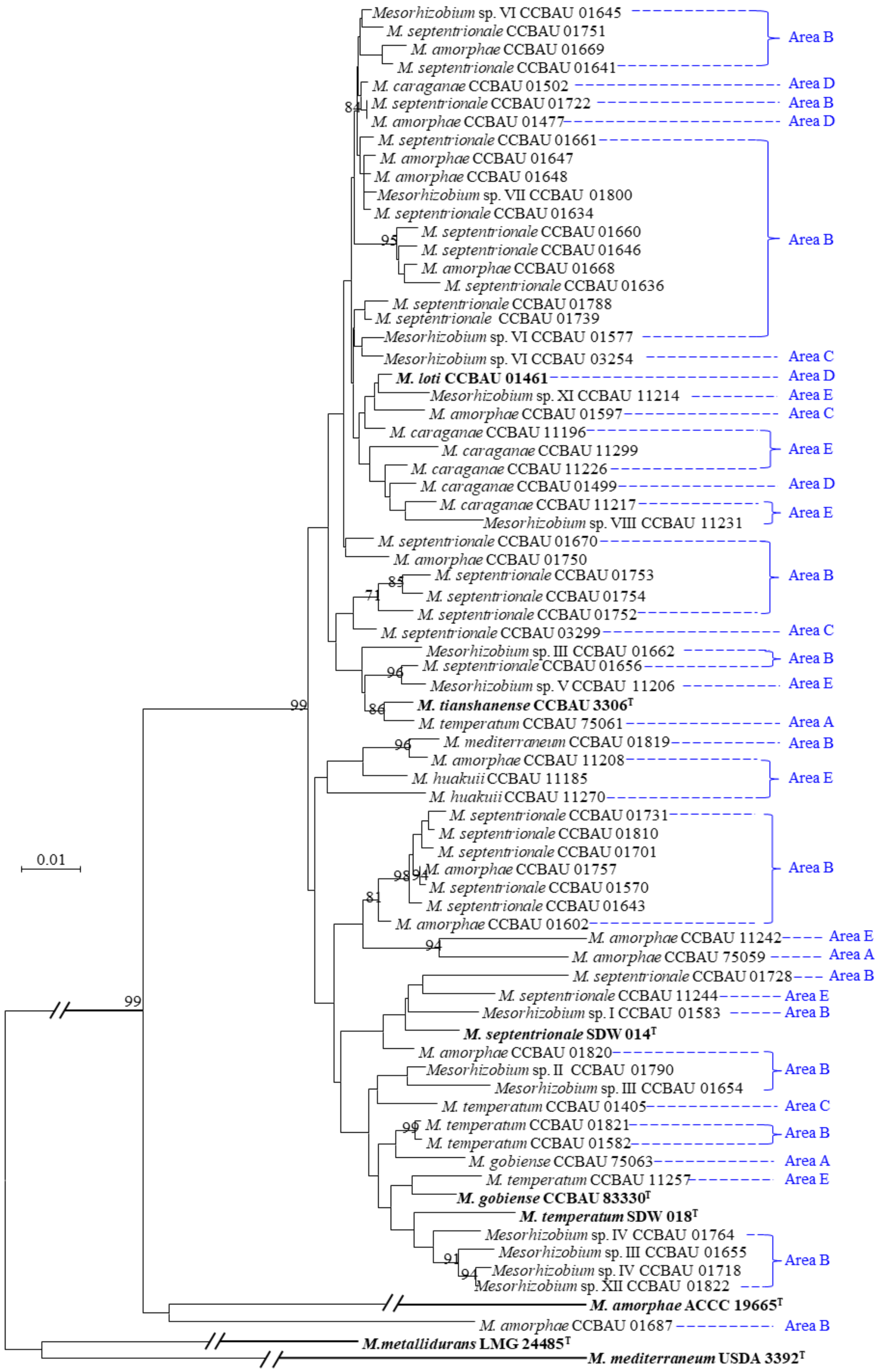

Supplementary Fig. S2. Neighbor-Joining (NJ) tree constructed based upon the concatenated sequences of nodulation genes. Two nodulation genes (*nodC*, *nodD*) were used. Bootstrap values greater than 70% are indicated at the branch points. The type strains were in bold fonts. The scale bar represents 1% nucleotide substitutions.

Supplementary Table S1. A total of 68 representative strains isolated from *Caragana*-associated *Mesorhizobium* used in this study.

Strains (CCBAU No.)	Species	Area	Collector	Province	City	County	Latitude	Longitude	pH	Ntot (g/Kg)	OM (g/Kg)	P (mg/Kg)	K (mg/Kg)
75059	*M. amorphae*	A	Li Mao	Ningxia	Wuzhong	Wuzhong	38.1272	105.9175	8.40	0.41	9.04	8.11	74.4
75063	*M. temperatum*	A	Li Mao	Ningxia	Zhongwei	Shapotou	37.4511	105.0230	8.43	0.37	9.76	4.89	84.4
75061	*M. gobiense*	A	Li Mao	Ningxia	Dujun	Gantangzheng	37.4261	104.6258	8.99	0.18	2.10	2.48	52.2
01570	*M. septentrionale*	B	Li Mao	Inner Mongolia	Ordos	Yangjiapo	39.7522	110.1211	8.73	0.53	7.14	6.10	60.8
01602	*M. amorphae*	B	Li Mao	Inner Mongolia	Ordos	Yangjiapo	39.7522	110.1211	8.73	0.53	7.14	6.10	60.8
01583	*Mesorhizobium* sp. I	B	Li Mao	Inner Mongolia	Ordos	Talahao	39.7892	110.1294	8.88	0.20	1.58	6.50	52.4
01577	*Mesorhizobium* sp. VI	B	Li Mao	Inner Mongolia	Ordos	Talahao	39.7892	110.1294	8.88	0.20	1.58	6.50	52.4
01582	*M. temperatum*	B	Li Mao	Inner Mongolia	Ordos	Talahao	39.7892	110.1294	8.88	0.20	1.58	6.50	52.4
01722	*M. septentrionale*	B	Zhaojun Ji	Inner Mongolia	Ordos	yijinhuoluoqi	39.1938	109.7915	8.62	0.29	4.64	7.71	67.0
01655	*Mesorhizobium* sp. III	B	Zhaojun Ji	Inner Mongolia	Ordos	yijinhuoluoqi	39.1938	109.7915	8.62	0.29	4.64	7.71	67.0
01757	*M. amorphae*	B	Zhaojun Ji	Inner Mongolia	Ordos	yijinhuoluoqi	39.1938	109.7915	8.62	0.29	4.64	7.71	67.0
01731	*M. septentrionale*	B	Zhaojun Ji	Inner Mongolia	Ordos	yijinhuoluoqi	39.1938	109.7915	8.62	0.29	4.64	7.71	67.0
01647	*M. amorphae*	B	Zhaojun Ji	Inner Mongolia	Ordos	yijinhuoluoqi	39.1938	109.7915	8.62	0.29	4.64	7.71	67.0
01790	*Mesorhizobium* sp. II	B	Zhaojun Ji	Inner Mongolia	Ordos	yijinhuoluoqi	39.1938	109.7915	8.62	0.29	4.64	7.71	67.0
01718	*Mesorhizobium* sp. IV	B	Zhaojun Ji	Inner Mongolia	Ordos	yijinhuoluoqi	39.1938	109.7915	8.62	0.29	4.64	7.71	67.0
01764	*Mesorhizobium* sp.IV	B	Zhaojun Ji	Inner Mongolia	Ordos	yijinhuoluoqi	39.1938	109.7915	8.62	0.29	4.64	7.71	67.0
01800	*Mesorhizobium* sp. VII	B	Zhaojun Ji	Inner Mongolia	Ordos	yijinhuoluoqi	39.1938	109.7915	8.62	0.29	4.64	7.71	67.0
01656	*M. septentrionale*	B	Zhaojun Ji	Inner Mongolia	Ordos	yijinhuoluoqi	39.1938	109.7915	8.62	0.29	4.64	7.71	67.0
01753	*M. septentrionale*	B	Zhaojun Ji	Inner Mongolia	Ordos	yijinhuoluoqi	39.1938	109.7915	8.62	0.29	4.64	7.71	67.0
01645	*Mesorhizobium* sp. VI	B	Zhaojun Ji	Inner Mongolia	Ordos	yijinhuoluoqi	39.1938	109.7915	8.62	0.29	4.64	7.71	67.0

Strains (CCBAU No.)	Species	Area	Collector	Province	City	County	Latitude	Longitude	pH	Ntot (g/Kg)	OM (g/Kg)	P (mg/Kg)	K (mg/Kg)
01810	*M. septentrionale*	B	Zhaojun Ji	Inner Mongolia	Ordos	yijinhuoluoqi	39.1938	109.7915	8.62	0.29	4.64	7.71	67.0
01634	*M. septentrionale*	B	Zhaojun Ji	Inner Mongolia	Ordos	yijinhuoluoqi	39.1938	109.7915	8.62	0.29	4.64	7.71	67.0
01662	*Mesorhizobium* sp. III	B	Zhaojun Ji	Inner Mongolia	Ordos	yijinhuoluoqi	39.1938	109.7915	8.62	0.29	4.64	7.71	67.0
01819	*M. mediterraneum*	B	Zhaojun Ji	Inner Mongolia	Ordos	yijinhuoluoqi	39.1938	109.7915	8.62	0.29	4.64	7.71	67.0
01701	*M. septentrionale*	B	Zhaojun Ji	Inner Mongolia	Ordos	yijinhuoluoqi	39.1938	109.7915	8.62	0.29	4.64	7.71	67.0
01660	*M. septentrionale*	B	Zhaojun Ji	Inner Mongolia	Ordos	yijinhuoluoqi	39.1938	109.7915	8.62	0.29	4.64	7.71	67.0
01687	*M. amorphae*	B	Zhaojun Ji	Inner Mongolia	Ordos	yijinhuoluoqi	39.1938	109.7915	8.62	0.29	4.64	7.71	67.0
01669	*M. amorphae*	B	Zhaojun Ji	Inner Mongolia	Ordos	yijinhuoluoqi	39.1938	109.7915	8.62	0.29	4.64	7.71	67.0
01646	*M. septentrionale*	B	Zhaojun Ji	Inner Mongolia	Ordos	yijinhuoluoqi	39.1938	109.7915	8.62	0.29	4.64	7.71	67.0
01788	*M. septentrionale*	B	Zhaojun Ji	Inner Mongolia	Ordos	yijinhuoluoqi	39.1938	109.7915	8.62	0.29	4.64	7.71	67.0
01820	*M. amorphae*	B	Zhaojun Ji	Inner Mongolia	Ordos	yijinhuoluoqi	39.8522	109.9011	8.62	0.29	4.64	7.71	67.0
01821	*M. temperatum*	B	Zhaojun Ji	Inner Mongolia	Ordos	yijinhuoluoqi	39.8522	109.9011	8.62	0.29	4.64	7.71	67.0
01643	*M. septentrionale*	B	Zhaojun Ji	Inner Mongolia	Ordos	yijinhuoluoqi	39.8522	109.9011	8.62	0.29	4.64	7.71	67.0
01728	*M. septentrionale*	B	Zhaojun Ji	Inner Mongolia	Ordos	yijinhuoluoqi	39.8522	109.9011	8.62	0.29	4.64	7.71	67.0
01739	*M. septentrionale*	B	Zhaojun Ji	Inner Mongolia	Ordos	yijinhuoluoqi	39.8522	109.9011	8.62	0.29	4.64	7.71	67.0
01670	*M. septentrionale*	B	Zhaojun Ji	Inner Mongolia	Ordos	yijinhuoluoqi	39.8522	109.9011	8.62	0.29	4.64	7.71	67.0
01822	*Mesorhizobium* sp. XII	B	Zhaojun Ji	Inner Mongolia	Ordos	yijinhuoluoqi	39.8522	109.9011	8.62	0.29	4.64	7.71	67.0
01668	*M. amorphae*	B	Zhaojun Ji	Inner Mongolia	Ordos	yijinhuoluoqi	39.8522	109.9011	8.62	0.29	4.64	7.71	67.0
01641	*M. septentrionale*	B	Zhaojun Ji	Inner Mongolia	Ordos	yijinhuoluoqi	39.8522	109.9011	8.62	0.29	4.64	7.71	67.0
01754	*M. septentrionale*	B	Zhaojun Ji	Inner Mongolia	Ordos	yijinhuoluoqi	39.8522	109.9011	8.62	0.29	4.64	7.71	67.0

Strains (CCBAU No.)	Species	Area	Collector	Province	City	County	Latitude	Longitude	pH	Ntot (g/Kg)	OM (g/Kg)	P (mg/Kg)	K (mg/Kg)
01654	*Mesorhizobium* sp. III	B	Zhaojun Ji	Inner Mongolia	Ordos	yijinhuoluoqi	39.8522	109.9011	8.62	0.29	4.64	7.71	67.0
01751	*M. septentrionale*	B	Zhaojun Ji	Inner Mongolia	Ordos	yijinhuoluoqi	39.8522	109.9011	8.62	0.29	4.64	7.71	67.0
01752	*M. septentrionale*	B	Zhaojun Ji	Inner Mongolia	Ordos	yijinhuoluoqi	39.8522	109.9011	8.62	0.29	4.64	7.71	67.0
01648	*M. amorphae*	B	Zhaojun Ji	Inner Mongolia	Ordos	yijinhuoluoqi	39.8522	109.9011	8.62	0.29	4.64	7.71	67.0
01661	*M. septentrionale*	B	Zhaojun Ji	Inner Mongolia	Ordos	yijinhuoluoqi	39.8522	109.9011	8.62	0.29	4.64	7.71	67.0
01750	*M. amorphae*	B	Zhaojun Ji	Inner Mongolia	Ordos	yijinhuoluoqi	39.8522	109.9011	8.62	0.29	4.64	7.71	67.0
01636	*M. septentrionale*	B	Zhaojun Ji	Inner Mongolia	Ordos	yijinhuoluoqi	39.8522	109.9011	8.62	0.29	4.64	7.71	67.0
01405	*M. temperatum*	C	Yangli Lu	Inner Mongolia	Wulancabu	Fengzhen	40.5583	113.3087	ND	ND	ND	ND	ND
01597	*M. amorphae*	C	Li Mao	Inner Mongolia	Ordos	Qingshuihe	39.9528	111.6767	ND	ND	ND	ND	ND
03299	*M. amorphae*	C	Yangli Lu	Shanxi	Xinzhou	Pianguan	39.4646	111.6719	ND	ND	ND	ND	ND
03254	*Mesorhizobium* sp. VI	C	Yangli Lu	Shanxi	Xinzhou	Pianguan	39.4646	111.6719	ND	ND	ND	ND	ND
01499	*M. caraganae*	D	Yangli Lu	Inner Mongolia	Xilinguole	Duolun	42.1966	116.4986	ND	ND	ND	ND	ND
01502	*M. caraganae*	D	Yangli Lu	Inner Mongolia	Chifeng	Balinyouqi	43.6848	118.9461	ND	ND	ND	ND	ND
01477	*M. amorphae*	D	Yangli Lu	Inner Mongolia	Xilinguole	Sanggendalai	42.6823	115.9453	ND	ND	ND	ND	ND
01461	*M. loti*	D	Yangli Lu	Inner Mongolia	Xilinguole	Sanggendalai	42.6823	115.9453	ND	ND	ND	ND	ND
11185	*M. huakuii*	E	Xuerui Yan	Liaoning	Fuxin	Zhangwu	42.5238	122.4742	ND	ND	ND	ND	ND
11196	*M. caraganae*	E	Xuerui Yan	Liaoning	Fuxin	Zhangwu	42.5238	122.4742	ND	ND	ND	ND	ND
11206	*Mesorhizobium* sp. V	E	Xuerui Yan	Liaoning	Yingkou	Dashiqiao	40.6469	122.5716	ND	ND	ND	ND	ND
11208	*M. septentrionale*	E	Xuerui Yan	Liaoning	Yingkou	Dashiqiao	40.6469	122.5716	ND	ND	ND	ND	ND
11214	*Mesorhizobium* sp. XI	E	Xuerui Yan	Liaoning	Yingkou	Dashiqiao	40.6469	122.5716	ND	ND	ND	ND	ND

Strains (CCBAU No.)	Species	Area	Collector	Province	City	County	Latitude	Longitude	pH	Ntot (g/Kg)	OM (g/Kg)	P (mg/Kg)	K (mg/Kg)
11217	*M. caraganae*	E	Xuerui Yan	Liaoning	Yingkou	Dashiqiao	40.6469	122.5716	ND	ND	ND	ND	ND
11226	*M. caraganae*	E	Xuerui Yan	Liaoning	Yingkou	Gaixian	40.6687	122.2333	ND	ND	ND	ND	ND
11231	*Mesorhizobium* sp. VIII	E	Xuerui Yan	Liaoning	Yingkou	Gaixian	40.6687	122.2333	ND	ND	ND	ND	ND
11242	*M. amorphae*	E	Xuerui Yan	Liaoning	Chaoyang	Jianping	41.3876	119.6203	ND	ND	ND	ND	ND
11244	*M. septentrionale*	E	Xuerui Yan	Liaoning	Chaoyang	Jianping	41.3876	119.6203	ND	ND	ND	ND	ND
11257	*M. temperatum*	E	Xuerui Yan	Liaoning	Chaoyang	Jianping	41.3876	119.6203	ND	ND	ND	ND	ND
11270	*M. huakuii*	E	Xuerui Yan	Liaoning	Tieling	Kaiyuan	42.2859	124.2259	ND	ND	ND	ND	ND
11299	*M. caraganae*	E	Xuerui Yan	Liaoning	Chaoyang	Beipiao	41.9360	121.1302	ND	ND	ND	ND	ND

Note: CCBAU, Culture Collection of Beijing Agricultural University. ND, undetermined. Values of pH, Ntot (g/Kg), OM (g/Kg), P (mg/Kg), K (mg/Kg) were the results of previous research (Li et al., 2012).

Supplementary Table S2. Primers designed for the genes and the annealing temperature of them (Tm, ℃) in PCR amplification.

**Genes**	**Complete name or function**	**primers (5'→3', forward/reverse)**	**Tm (℃)**	**References[#]**
recA	Recombinase A	TTCGGCAAGGGMTCGRTSATG/ ACATSACRCCGATCTTCATGC	54	(Ji et al., 2015)
rpoB	DNA-directed RNA polymerase subunit beta	ATCGTCTCGCAGATGCACCG/ TCGATGTCGTCGATYTCGCC	58	(Ji et al., 2015)
nodC	N-acetylglucosaminyltransferase, NodC	TGATYGAYATGGARTAYTGGCT/ CGYGACARCCARTCGCTRTTG	52	(Ji et al., 2015)
nodD	Transcriptional regulator, NodD	GCGAACGYWTTCTGACACC/ TAAATSCSGGAAGTGGC	55	(Ji et al., 2015)
cysW	sulfate/thiosulfate ABC transporter inner membrane subunit	GCCGCTGATCRTCGTYTT/ CGCCAYTGCCYTGYTCC	57	This study
exoY	exopolysaccharide production protein ExoY	CCSTGCCTSAARTTCCG/ ARCGACCAGTTYTCGACATARTG	62	This study
idhA	myo-inositol dehydrogenase	CACSATCCGCAACATYCT/ CCGTARTTSAGCGARGC	58	This study
mutS	DNA mismatch repair protein MutS	TGGGCGATTTCTACGAG/ TCSGCYTTYTGCGTYTT	60	This study
uvrC	excinuclease ABC subunit UvrC	AAGAAGCGBGTSACYAAYTAC/ GAAYTGCGCCAGRAACG	50	This study

[#] Primers for some genes were designed referring to the corresponding homologous regions of the whole genomes (unpublished) of *M. amorphae* CCBAU 01578, *M. silamurunense* CCBAU 01550^{T}, *M. mediterraneum* CCBAU 01399 and *M. caraganae* CCBAU 01502 using the Primer 5.0 software in this study.

Supplementary Table S3. Shimodaira-Hasegaewa (SH) test of each test gene locus in comparison with the concatenated core genes.

**Genes**	**-ln *L***	**Diff -ln *L***	***P***
cysW	19434.52	1482.48	0.004*
exoY	19726.40	1774.35	0.000*
idhA	20191.31	2239.26	0.000*
mutS	20034.85	2082.81	0.000*
uvrC	21315.33	3363.28	0.000*

Note: -ln *L*, negative log-likelihood value for the constrained topology; Diff –ln *L*, score difference between the nonconstrained and constrained trees; *P*, significance of the difference in –ln *L* scores calculated based on the constrained and unconstrained trees as assessed by the SH test. *, $P < 0.05$.

Supplementary Table S4. Accession numbers of the genes obtained in this study and deposited in GenBank.

Genes	GenBank Accession numbers
**Membrane-transporter genes**	
cysW	MK252304 - MK252371
exoY	MK252372 - MK252439
idhA	MK252440 - MK252507
**Nucleotide repair genes**	
mutS	MK252508 - MK252575
uvrC	MK252576 - MK252643

References

Li, M., Y. Li, W. F. Chen, X. H. Sui, L. Y. Jr, Y. Li, E. T. Wang, and W. X. Chen. 2012. Genetic diversity, community structure and distribution of rhizobia in the root nodules of *Caragana* spp. from arid and semi-arid alkaline deserts, in the north of China. *Systematic and Applied Microbiology*, 35(4): 239-45. https:// doi: 10.1016/j.syapm.2012.02.004.

Ji, Z. J., Z. J. Ji, Q. G. Cui, E. T. Wang, W. X. Chen, and W. F. Chen. 2015. "Genetic Divergence and Gene Flow among *Mesorhizobium* Strains Nodulating the Shrub Legume *Caragana*". *Systematic and Applied Microbiology*, 38(3), 176-183. https://doi:10.1016/j.syapm.2015.02.007
